# A retrospective analysis of survival and prognostic factors after stereotactic radiosurgery for aggressive meningiomas

**DOI:** 10.1186/1748-717X-9-38

**Published:** 2014-01-27

**Authors:** Daniel J Ferraro, Ryan K Funk, John William Blackett, Michelle R Ju, Todd A DeWees, Michael R Chicoine, Joshua L Dowling, Keith M Rich, Robert E Drzymala, Imran Zoberi, Joseph R Simpson, Jerry J Jaboin

**Affiliations:** 1 Department of Radiation Oncology, Washington University in Saint Louis, 4511 Forest Park Avenue/Campus Box 8224, St. Louis, MO, USA; 2 Department of Radiation Oncology, Mayo Clinic, Rochester, MN, USA; 3 School of Medicine, University of Washington, Seattle, WA, USA; 4 Department of Neurological Surgery, Washington University Medical School, Saint Louis MO, USA

**Keywords:** Atypical, Anaplastic, Aggressive, WHO II, WHO III, Gamma knife, Radiosurgery, Meningioma

## Abstract

**Background:**

While most meningiomas are benign, aggressive meningiomas are associated with high levels of recurrence and mortality. A single institution’s Gamma Knife radiosurgical experience with atypical and malignant meningiomas is presented, stratified by the most recent WHO classification.

**Methods:**

Thirty-one patients with atypical and 4 patients with malignant meningiomas treated with Gamma Knife radiosurgery between July 2000 and July 2011 were retrospectively reviewed. All patients underwent prior surgical resection. Overall survival was the primary endpoint and rate of disease recurrence in the brain was a secondary endpoint. Patients who had previous radiotherapy or prior surgical resection were included. Kaplan-Meier and Cox proportional hazards models were used to estimate survival and identify factors predictive of recurrence and survival.

**Results:**

Post-Gamma Knife recurrence was identified in 11 patients (31.4%) with a median overall survival of 36 months and progression-free survival of 25.8 months. Nine patients (25.7%) had died. Three-year overall survival (OS) and progression-free survival (PFS) rates were 78.0% and 65.0%, respectively. WHO grade II 3-year OS and PFS were 83.4% and 70.1%, while WHO grade III 3-year OS and PFS were 33.3% and 0%. Recurrence rate was significantly higher in patients with a prior history of benign meningioma, nuclear atypia, high mitotic rate, spontaneous necrosis, and WHO grade III diagnosis on univariate analysis; only WHO grade III diagnosis was significant on multivariate analysis. Overall survival was adversely affected in patients with WHO grade III diagnosis, prior history of benign meningioma, prior fractionated radiotherapy, larger tumor volume, and higher isocenter number on univariate analysis; WHO grade III diagnosis and larger treated tumor volume were significant on multivariate analysis.

**Conclusion:**

Atypical and anaplastic meningiomas remain difficult tumors to treat. WHO grade III diagnosis and treated tumor volume were significantly predictive of recurrence and survival on multivariate analysis in aggressive meningioma patients treated with radiosurgery. Larger tumor size predicts poor survival, while nuclear atypia, necrosis, and increased mitotic rate are risk factors for recurrence. Clinical and pathologic predictors may help identify patients that are at higher risk for recurrence.

## Background

Meningiomas are tumors of the meninges, the membranes overlying the brain and spinal cord. They are derived from arachnoid cap cells, which are cytologically similar to meningioma tumor cells [1,2]. Intracranial meningiomas account for 24-33% of primary brain tumors [3,4] and have an incidence rate of 6 per 100,000 people per year [4]. In the absence of genetic or environmental risk factors, meningiomas occur primarily in the 6th to 8th decades of life [1,5,6]. Meningiomas are more common in females by a ratio of 2:1 [7]. Common presenting symptoms include headache and seizures.

Meningiomas are classified by the World Health Organization based on morphology on a scale of I-III. Eighty to ninety percent are benign (WHO Grade I). Five to twenty percent are atypical (WHO Grade II) and 1-5% are anaplastic or malignant (WHO Grade III) [8-12].

WHO grade II and III tumors are considered aggressive. While relatively rare, they are much more difficult to treat than benign meningiomas. According to current guidelines, WHO grade II meningiomas have increased mitotic activity (≥4 mitoses per high powered field), or at least three of the following traits: prominent nucleoli, increased cellularity, small cells with a high nuclear to cytoplasmic ratio, foci of spontaneous or geographic necrosis, and uninterrupted patternless or sheet-like growth. Atypical, chordoid, and clear cell meningiomas are also classified as WHO grade II. WHO grade III meningiomas have ≥20 mitoses/high-powered field and/or malignant characteristics resembling those of sarcoma, melanoma, or carcinoma. Papillary, anaplastic, and rhabdoid meningiomas are also classified as WHO grade III.

Five- and ten-year overall survival rates for all meningiomas are high at 82% and 64% respectively [1], but prognosis for aggressive meningiomas is much worse [13,14]. Survival rates for patients with aggressive meningiomas are 65% at five years and 51% at ten years [15]. Grade II and III tumors are more likely to recur and are associated with worse overall survival. Compared to age and sex matched benign meningiomas, grade II meningiomas are approximately 8 times more likely to recur [16]. Furthermore, recurrent meningiomas tend to be more aggressive than the original [17]. Aggressive meningiomas are more common among men than women [18].

Adjuvant radiosurgery is increasingly being used after surgery in the treatment of meningiomas, although a recent study of 228 atypical meningiomas found no difference in recurrence rates between patients treated with adjuvant radiosurgery, adjuvant intensity modulated radiation therapy, and surgery alone [19].

Limited data is available regarding treatment outcomes for patients with aggressive meningiomas treated with radiosurgery. Additionally, much of the data regarding treatment for aggressive meningiomas is based on pre-2000 series. Further information regarding patient characteristics and outcomes is necessary to better guide management decisions for these patients.

This study reviews the outcomes of 31 grade II and 4 grade III meningioma patients treated with Gamma Knife radiosurgery at Washington University in St. Louis. Patient characteristics and previously reported prognostic factors for disease-free and overall survival are also analyzed.

## Methods

### Patient selection

Patients with a prior pathologic diagnosis of WHO grade II–III meningioma treated with Gamma Knife radiosurgery at Washington University Medical Center between July 2000 and July 2011 were retrospectively reviewed. Our Gamma Knife Unit is a shared community resource open to qualified neurosurgeons and radiation oncologists from the greater Saint Louis area. This review was restricted to patients treated by the neurosurgical and radiation oncology faculty of the Washington University School of Medicine. There were no other exclusions. The main endpoint of the study was overall survival; disease recurrence in the brain was a secondary endpoint. Patients who had previous radiotherapy or prior surgical resection were included. Patients who underwent surgical resection were medically stable at the time of surgery and believed to have life expectancies greater than 6 months. Surgical resection was classified by the neurosurgeon performing the resection as either a gross-total resection (GTR) or subtotal resection (STR), or biopsy only at the time of surgery. WHO Classification is consistent with the most recent WHO 2007 Classification. Patients were followed serially in both the Neurosurgical and Radiation Oncology clinics. Washington University School of Medicine Human Research Protection Office reviewed and approved this study, IRB ID# 201010707.

### Radiosurgical technique

Stereotactic radiosurgery was performed at the Gamma Knife of Saint Louis facility at Barnes Jewish Hospital/Washington University Medical Center. All patients underwent stereotactic radiosurgery using a Gamma Knife unit (Elekta, Atlanta, GA). From 1998 to August 2002 a Model B was in operation, while from August 2002 to April 2008 a Model C was used. Currently a Perfexion unit is in use. All patients had intravenous access placed. A Leksell frame was placed under local anesthesia often with a low, intravenous dose of an anxiolytic. In most cases, a contrast enhanced computed tomography scan and a contrast enhanced magnetic resonance image were obtained and images transferred to the GK treatment-planning computer. The target(s) were contoured and a radiosurgery plan was developed. Tumor size, tumor location, and history of prior radiation therapy were important factors in selection of the prescription dose. The prescription dose was typically prescribed to the 50% isodose line, which followed the tumor margin. Quality Assurance review consisted of the following: verification of patient name from all imaging; verification of patient orientation (visible surface markers on the patient’s left side and patient anatomy); review of MR fiducial alignment; verification of patient identity (patient name and birth date by all members of the treatment team); as well as verification of the treatment specifications at the console after transfer by comparison against the paper treatment plan.

The medical record was retrospectively reviewed to determine pretreatment patient and tumor characteristics and the dates of time-to-event endpoints. The recurrence of treated disease, progression of known disease, or the development of new brain metastasis was scored as a failure for brain recurrence. Death from any cause was a failure for overall survival. All times were measured from the date of radiosurgery.

### Statistical analysis

Standard measures of central tendency and dispersal were used to characterize patient and tumor parameters. Survival time and time to CNS recurrence were calculated from the date of SRS. The Kaplan-Meier method was used to describe time to brain recurrence and overall survival. The log-rank test was used to compare these endpoints. The Cox Proportional Hazards method was used to identify predictors of overall survival. Based on published literature, several known predictors such as age, performance status, and presence of extra-CNS disease were force-entered into the model. Suspected predictive factors including treatment intent and number of brain metastases were then entered using backwards deletion. Statistical tests were performed using SAS version 9.2 (SAS Institute Inc., Cary, NC). All statistical tests were two-sided with p values < 0.05 deemed significant.

## Results

### Patient characteristics

Thirty-one patients with atypical and four patients with malignant meningiomas treated with Gamma Knife stereotactic radiosurgery between 2000 and 2011 were reviewed. All patients received surgery as their first treatment modality. A summary of patient characteristics is presented in Table 1. Eighteen of the patients (51.4%) were male. The median patient age was 57 years (range: 26 – 81 years) at surgery, and 61 years (range: 26 – 84 years) at the time of radiosurgery. Seven tumors were located in the skull base, 16 along the convexity, 11 in the parasagittal/falcine region, and 1 intraventricular tumor.

**Table 1:** Patient characteristics

**Characteristic**	**Median (range) or n(%)**	
Age at surgery	57 (26–81)	
Age at radiosurgery	61 (26–84)	
Death	9 (25.7%)	
Recurrence	11 (31.4%)	
Sex		
Female	17 (48.6%)	
Male	18 (51.4%)	
Histology		
WHO Grade II	31 (88.6%)	
WHO Grade III	4 (11.4%)	
Risk factors		
Hypertension	9 (25.7%)	
Diabetes mellitus	0 (0%)	
Rheumatoid arthritis	0 (0%)	
Lupus	0 (0%)	
Presenting signs and symptoms		
Headache	10 (28.6%)	
Seizure	6 (17.1%)	
Other neurological finding	18 (51.4%)	
Incidental	1 (2.9%)	
Type of surgical resection		
Gross total	18 (51.4%)	
Partial	14 (40%)	
Unknown	2 (5.7%)	
Prior malignancy		
Yes	3 (8.6%)	
No	28 (80%)	
Pre-diagnosis cranial radiation	2 (5.7%)	
Margin dose	18 Gy (14–24)	
Tumor characteristics		
Tumor volume	3.90 cm^3^ (0.19-33.1)	
Treated tumor volume	3.90 cm^3^ (0.19-31.9)	
History of previous benign meningioma	3 (8.6%)	

### Radiosurgical parameters

All patients were treated as described in the methods section. The median tumor volume was 3.90 cm^3^ (range: 0.19-33.1), as was the median treated tumor volume (range: 0.19-31.9). The median radiosurgical margin dose was 18 Gy (range: 14–24 Gy) with a median of 10 isocenters (range: 2–49). The median time from surgery to Gamma Knife radiosurgery was 21.1 months (range: 0.1 – 246 months), and the median follow-up time post-Gamma Knife was 34.5 months (range: 0 – 117 months).

### General patient survival outcomes

Kaplan-Meier and Cox proportional hazards models were used to estimate survival curves and examine the association between clinical, pathologic, and imaging parameters for recurrence and overall survival. Post-Gamma Knife recurrence was identified in 11 patients, and 9 patients had died. The median time to recurrence from Gamma Knife was 25.8 months (range: 0 – 113 months) and the median overall survival was 36 months (range: 0 – 120 months). One and three-year overall survival (OS) rates were 86.1% and 78.0% respectively. One and three-year progression-free survival (PFS) rates were 88.7% and 65.0%, respectively.

### Patient outcomes based on WHO grade

**Figure 1:**
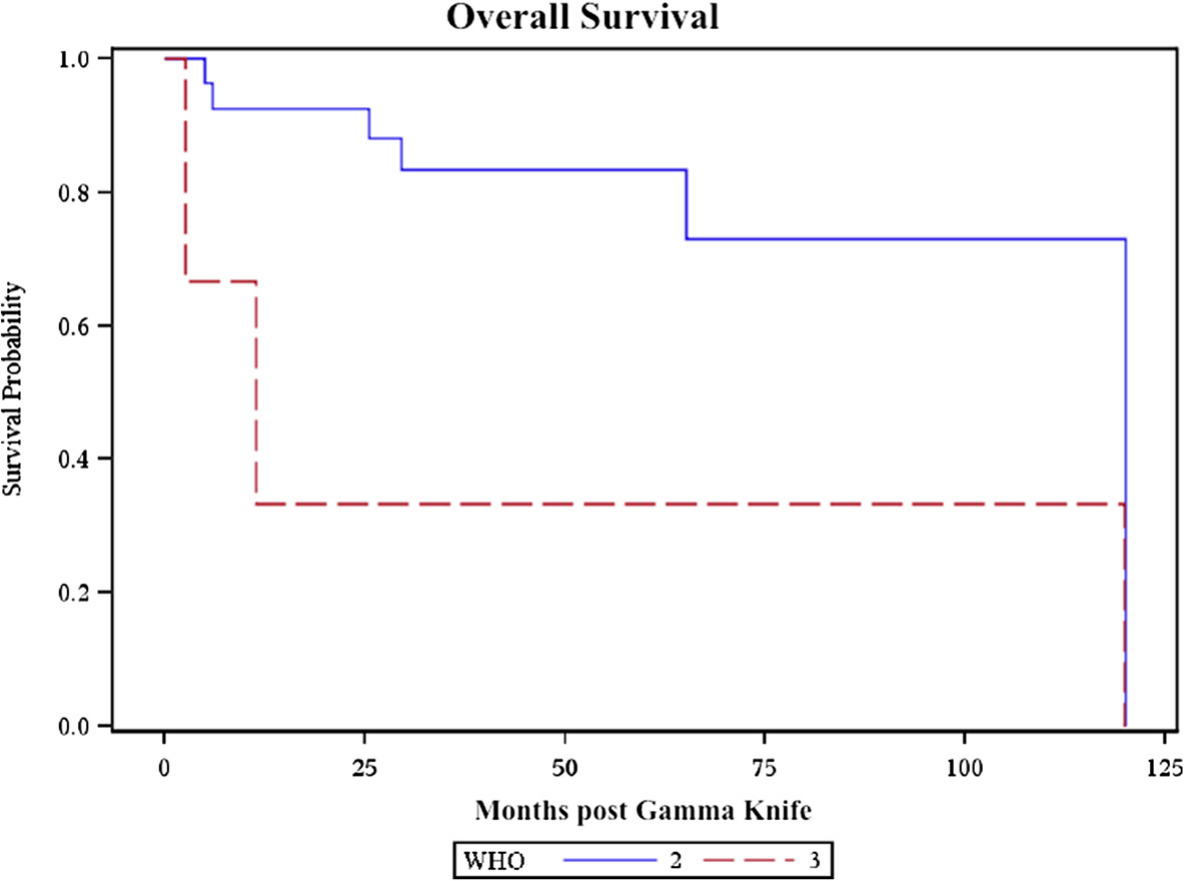
**Overall survival.** Kaplan-Meier overall survival curve of WHO grade II (blue line) and grade III (red line) patients in months post-Gamma Knife.

**Figure 2:**
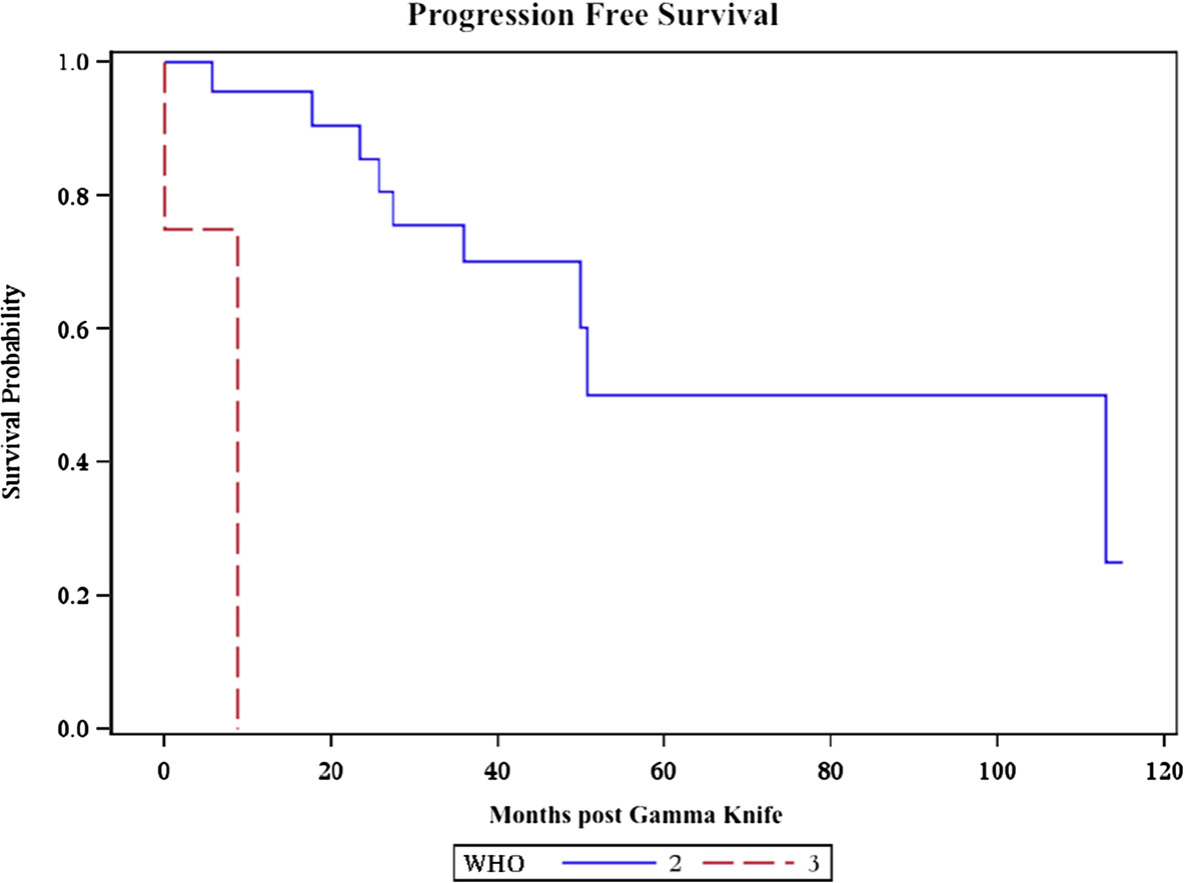
**Progression-free survival.** Kaplan-Meier progression-free survival curve of WHO grade II (blue line) and grade III (red line) patients in months post-Gamma Knife.

To analyze the effect of WHO grade on outcomes, atypical grade II meningiomas were compared with malignant grade III meningiomas. WHO grade II 1 and 3-year OS rates were 92.4% and 83.4% respectively. WHO grade II 1 and 3-year PFS rates were 95.7% and 70.1% respectively. WHO grade III 1 and 3-year OS rates were both 33.3%. WHO grade III 1 and 3-year PFS rates were both 0%. WHO grade III histology was significantly associated with decreased survival (HR: 5.52, *p* = 0.031) and increased recurrence (HR: 31.9, *p* = 0.0057). The overall survival curves of grade II and III patients are presented in Figure 1; the progression-free survival curves of grade II and III patients are presented in Figure 2.

### Patient outcomes based on known prognostic parameters

A summary of parameters significant for recurrence or survival is presented in Table 2.

**Table 2:** Significant prognostic factors for overall survival and recurrence

**Overall survival**			**Recurrence**		
**Parameter**	**Hazard ratio**	**p value**	**Parameter**	**Hazard ratio**	**p value**
Prior history of benign meningioma	10.46	0.02075	Prior history of benign meningioma	24.9765	0.00938
Prior fractionated RT	5.76	0.0234	Necrosis	5.4325	0.03085
WHO grade III	5.52	0.03137	WHO grade III	31.8875	0.0057
Time from surgery to GK	1.01	0.01902	Nuclear atypia	4.6286	0.015
Isocenter volume	1.04	0.01058	Margin dose	1.0061	0.00051
Tumor volume treated	1.08	0.00824	Mitotic rate	1.1802	0.00742
Tumor volume	1.08	0.00812			
Isocenter number	1.17	0.00146			

The histopathology of meningiomas was analyzed to identify parameters that significantly impacted survival and recurrence. Of the characteristics used to diagnose meningiomas as aggressive, including high nuclear to cytoplasmic ratio, increased cellularity, prominent nucleoli, spontaneous or geographic necrosis, sheet-like growth, nuclear atypia, and increased mitotic rate, only necrosis, nuclear atypia, and mitotic rate were significantly predictive. On univariate analysis, the presence of nuclear atypia in 11 patients was associated with increased recurrence (HR: 4.63, *p* = 0.0150), as was spontaneous necrosis in 17 patients (HR: 5.43, *p =* 0.0309) and increasing mitotic rate (HR: 1.18, *p* = 0.00742).

Parameters relating to patients’ medical histories that affected their survival were identified using univariate analysis. A prior history of benign meningioma in 3 patients was associated with both worse overall survival (HR: 10.46, *p* = 0.0208) and increased recurrence (HR: 24.98, *p* = 0.00938). A prior history of fractionated radiation therapy in 9 patients was associated with worse overall survival (HR: 5.76, *p* = 0.0234). Increasing length of time between surgery and Gamma Knife significantly decreased overall survival (HR: 1.01, *p* = 0.0190), although the clinical effect was small.

Parameters relating to the radiosurgery itself that significantly affected survival were also examined. Increasing margin dose of radiation was significantly associated with recurrence (HR: 1.01, *p* = 0.00051), although again the clinical effect was small. Overall survival was detrimentally impacted by both higher tumor volume (HR: 1.08, *p* = 0.00812) and treated tumor volume (HR: 1.08, *p* = 0.00824). Increasing the number of isocenters (HR 1.17, *p* = 0.00146) and the volume of each isocenter (HR: 1.04, *p* = 0.0106) adversely affected overall survival.

Age at surgery and at the time of radiosurgery did not have a significant impact on either survival or recurrence in this cohort. The presence of bone or brain invasion did not have a significant impact on either survival or recurrence, possibly because our cohort only included one instance of bone invasion and seven of brain invasion.

On multivariate analysis, the only significant factor for recurrence was WHO grade II histology (HR: 0.047, p = 0.0134), while the absence of spontaneous necrosis had a trend towards increased survival (HR: 0.217, p = 0.0556). The only significant factors for overall survival on multivariate analysis were WHO grade II histology (HR: 0.168, p = 0.0386) and high treated tumor volume (HR: 1.096, p = 0.0095).

### Analysis of atypical (WHO grade II) meningiomas

In order to better identify prognostic factors of atypical meningiomas, an additional analysis of only the 31 WHO grade II patients was carried out. Significant prognostic parameters are presented in Table 3. The median time to recurrence post-Gamma Knife for this cohort was 27.5 months. The median survival post-Gamma Knife was 36.2 months.

**Table 3:** Significant prognostic factors in WHO grade II meningiomas alone

**Overall survival**			**Recurrence**		
**Parameter**	**Hazard ratio**	**p value**	**Parameter**	**Hazard ratio**	**p value**
Prior fractionated RT	6.31	0.04572	Bone invasion	16.43	0.04801
Time from surgery to GK	1.01	0.02578	Pre-event RT	9.52	0.01157
Isocenter number	1.18	0.01361	Margin dose	1.01	0.00151
Isocenter volume	1.05	0.01222			
Tumor volume	1.09	0.00889			
Treated tumor volume	1.1	0.00879			

On univariate analysis, the parameters significant for recurrence in this cohort were bone invasion (HR: 16.43, *p* = 0.0480), pre-event radiation therapy (HR: 9.52, *p* = 0.0116), and margin dose (HR: 1.01, *p =* 0.00151). On multivariate analysis, margin dose was the only significant parameter for recurrence (HR: 1.01, p = 0.0065).

The parameters significant for overall survival on univariate analysis were a history of prior fractionated RT (HR: 6.31, *p* = 0.0457), time from surgery to GK (HR: 1.01, *p* = 0.0258), isocenter number (HR: 1.18, *p* = 0.0136) and isocenter volume (HR: 1.05, *p* = 0.0122), tumor volume (HR: 1.09, *p* = 0.00889), and treated tumor volume (HR: 1.10, *p* = 0.00879). On multivariate analysis, only treated tumor volume was significant for overall survival in this group (HR: 1.01, p = 0.0088).

## Discussion

WHO grade II and III meningiomas are rare and heterogeneous tumors of the meninges. Their outcomes and rates of recurrence can be difficult to predict. This complicates the selection of an appropriate therapy. More precise and accurate prognostic factors are needed in order to identify tumors associated with recurrence and decreased survival. Previous studies of WHO grade II and III meningiomas have identified age, sex, WHO grade, tumor location, and extent of resection as useful prognostic parameters [9]. Table 4 presents previously reported outcomes of aggressive meningiomas treated by Gamma Knife radiosurgery.

**Table 4:** Literature review of aggressive meningiomas treated by gamma knife

**Author, year**	**WHO grade**	**Patient number (lesions)**	**Marginal dose (Gy)**	**Max dose (Gy)**	**Control rate**	**Progression free survival**	**Overall survival**	**Statistically significant prognostic factors on multivariate analysis**
Ojemann et al., 2000 [20]	3	19 (31)	Mean 16	18		5 year 26%	5 year 40%	PFS and OS: age (p < 0.003), tumor volume (p < 0.05)
Stafford et al., 2001 [21]	2	13	Median 16	36	5 year 68%		5 year 76%*	
	3	9			5 year 0%		5 year 0%*	
Harris et al., 2003 [22]	2	18	Mean 14.9	29.4		5 year 83%	5 and 10 year 59%	PFS: early SRS (p < 0.001), small tumor volume (p < 0.001)
	3	12	Mean 15.7	31.4		5 year 72%	5 year 59% 10 year 0%	OS: younger age (p = 0.03)
Huffmann et al., 2005 [23]	2	15 (21)		18	6 months 93%		100% at median follow up of 35 months	
Kondziolka et al., 2008 [24]	2	54			50% at median of 2 years			
	3	29			17% at median of 15 months			
Attia et al., 2012 [25]	2	24	Median 14	18	1 year 75%	2 year 40%	1 year 92%	PFS: dose > 14 Gy (p = 0.01)
					2 year 51%	5 year 25%	2 year 67%	
					5 year 44%		5 year 52%	
Mori et al., 2013 [26]	2	19 (22)	Mean 16.5	20.15	1 year 74%,			
	3	4						
					2 year 54%,			
					3 year 34%			
Tamura et al., 2013 [27]	2	9	Mean 18.8	37				Stabilization of tumor growth: small lesion volume (p = 0.02), marginal dose (p = 0.04), max dose (p = 0.02)
	3	7						
Current series	2	31	Median 18	24		1 year 95.7% 3 year 70.1%	1 year 92.4% 3 year 83.4%	Recurrence: WHO grade 2 histology (p = 0.0134)
	3	4				1 and 3 year 0%	1 and 3 year 33.3%	OS: WHO grade 2 histology (p = 0.0386), treated tumor volume (p = 0.0095)

*Cause specific survival.

The current study analyzed 35 patients with atypical (WHO grade II) and malignant (WHO grade III) meningiomas who received Gamma Knife radiosurgery between 2000 and 2011. The purpose was to identify characteristics that predict survival and recurrence, as well as to determine the overall and progression-free survival of patients with aggressive meningiomas treated with radiosurgery at our institution.

WHO grade is a well-known prognostic factor from previous studies. Grade III meningiomas are typically associated with poorer survival and higher rates of recurrence [9,28]. However, a 2008 series of 119 grade II and III meningioma patients treated with external beam radiotherapy reported no significant differences in overall survival or disease-free survival based on WHO grade [15]. Kondziolka et al. demonstrated that radiosurgery produces effective local control in benign meningiomas, but had worse outcomes for aggressive tumors. They reviewed 384 grade I tumors and found a tumor control rate of 93% at a median of 4 years. They found a 50% control rate in 54 grade II tumors at a median of 2 years, and a 15% control rate in 29 grade III tumors at a median of 15 months [24].

A 2009 series of 199 aggressive meningioma patients treated surgically found the 5- and 10- year OS rates of grade II patients were 78.4% and 53.3% respectively [9]. The OS rates of grade III patients were 44.0% and 14.2% respectively. The 5- and 10-year PFS rates for grade II were 48.4% and 22.6% respectively, while for grade III they were 8.4% and 0.0%.

In another analysis of aggressive meningiomas, Mori et al. looked at 22 grade II and 4 grade III meningioma patients undergoing Gamma Knife radiosurgery [26]. They found local tumor control after treatment was 74% at 1 year, 52% at 2 years, and 34% at 3 years. Attia et al. analyzed 24 patients with atypical meningiomas treated with Gamma Knife radiosurgery as either primary or salvage therapy and found local control rates at 1, 2, and 5 years of 75, 51, and 44% [25].

Among our patients, the 3-year OS rates were 84.1% for grade II and 33.3% for grade III. The 3-year PFS rates were 70.1% for grade II and 0.0% for grade III. These results agree with previous findings that WHO grade III tumors are associated with poorer outcomes, although there were only 4 grade III meningioma patients in our series. Three-year OS and PFS rates overall were 78.0% and 65.0%, respectively.

Histological features associated with aggressive meningiomas were also examined, such as hypercellularity, brain invasion, elevated mitotic rate, prominent nucleoli, and cell necrosis. Kim et al. found that anaplasia, mitotic index ≥20/10 high-power fields, subtotal tumor resection, loss of short arm of chromosome 1 (1p-), and Ki-67 labeling index >12% were independent predictors of recurrence [29]. Pasquier et al., in their 2008 series of 119 grade II and III meningiomas treated with external beam radiotherapy, also found that high mitotic rate adversely affected overall survival and disease-free survival [15]. In our study, high mitotic rate, spontaneous necrosis, and nuclear atypia were associated with increased rates of recurrence on univariate analysis.

A retrospective analysis of 35 patients with 49 atypical and anaplastic meningiomas treated with radiosurgery found local tumor control rates for grade II meningiomas at 1, 2 and 3 years after radiosurgery were 78, 53 and 36%, respectively [30]. Grade III meningioma local control rates were 35% at 1 year and 10% at 2 years. Multivariate analysis indicated that the mitotic count and the MIB-1 proliferation marker labeling index were significant prognostic factors.

We did not find prominent nucleoli, sheeting, high nucleus to cytoplasm ratio, hypercellularity, frank anaplasia, bone invasion, or brain invasion to be associated with either survival or recurrence, although bone invasion was significant for recurrence on univariate analysis among the WHO II meningiomas alone. This may be affected by the relatively low prevalence of bone invasion (1 instance) and brain invasion (7 instances) in this series, as well as the low number of grade III meningiomas.

Age under 60 at diagnosis has been identified as a predictor of better outcomes among aggressive meningioma patients [9,15]. We did not find either age at initial surgery or age at radiosurgery to be a significant parameter for survival or recurrence in our series.

Gross total resection has also been identified in previous studies as a favorable prognostic parameter [15,29]. In an analysis of 199 aggressive meningiomas treated surgically, Durand et al. found that age under 60, Simpson grade I resection, and grade II histology were independent prognostic factors for survival on multivariate analysis [9]. However our study did not find that the extent of resection was predictive of recurrence or overall survival.

The three patients in our series with a prior history of benign meningioma exhibited strong associations with recurrence (HR 25.0, p = 0.021) and poor survival (HR 10.5, p = 0.0094). This suggests that benign meningiomas may be more dangerous than realized, as patients who subsequently develop atypical or malignant meningiomas have a more aggressive course. We also found that patients who had undergone previous fractionated radiotherapy had worse overall survival on univariate analysis (HR: 5.76, p = 0.0234). This may simply reflect that these patients had a more aggressive or difficult course that therefore received more and earlier treatment.

An increasing length of time between surgery and Gamma Knife was statistically associated with worse overall survival, but the effect was so small (HR 1.01, p = 0.019) that this is unlikely to be clinically significant.

Tumor size was significantly associated with worse overall survival in our series. Larger tumor volumes and treated tumor volumes were both associated with poor survival, but not increased recurrence. Larger tumors are at a greater risk of herniation, leading to higher mortality rates. These results agree with other studies that have shown that among benign meningiomas treated with Gamma Knife, larger tumors are associated with significantly worse 5-year disease-free survival and tumor control [31,32]. Tamura et al. analyzed 9 grade II and 7 grade III meningiomas and found that small lesion volume, as well as greater marginal and maximum irradiation doses, were associated with stabilization of tumor growth [27].

The strongest association in our series was between WHO grade III histology and recurrence (HR 31.9, p = 0.0057). Interestingly, the individual histological features used to determine WHO grade were either not significant or considerably less significant. Nuclear atypia (HR 4.63, p = 0.015), necrosis (HR 5.43, p = 0.031), and high mitotic rate (HR 1.18, p = 0.0074) were significant for recurrence. The other histological features were not significant for either recurrence or survival. This suggests that it is the combination of individual features, rather than any one factor, that determines prognosis, and strengthens the credibility of the WHO grading criteria. WHO grade III meningiomas were also strongly associated with worse overall survival (HR 5.52, p = 0.031), although not to the same degree as recurrence. On multivariate analysis, WHO grade II diagnosis was the only significant favorable prognostic factor for recurrence (HR: 0.047, p = 0.0134). WHO grade II diagnosis (HR: 0.168, p = 0.0386) and larger tumor volume treated (HR: 1.096, p = 0.0095) were the only significant prognostic factors for decreased overall survival on multivariate analysis.

When analyzing WHO grade II meningiomas alone, the most significant parameters for overall survival were isocenter number, isocenter volume, tumor volume, and treated tumor volume, which were all highly correlated with each other and largely reflect tumor size. On multivariate analysis of WHO II tumors alone, increased tumor volume treated was the only parameter significant for decreased overall survival (HR 1.01 and p = 0.0088). A history of prior fractionated radiation therapy and increasing length of time between surgery and radiosurgery were also significantly associated with worse overall survival on univariate analysis.

Bone invasion (HR 16.43, p = 0.048) and pre-event radiation therapy (HR 9.52, p = 0.01157) were significantly associated with recurrence on univariate analysis among the WHO II tumors alone.

Increasing margin dose was significantly associated with increased recurrence among grade II meningioma patients (HR 1.01, p = 0.0015). It was also the only significant variable for recurrence on multivariate analysis (HR 1.01, p = 0.0065). This most likely reflects that the tumors at highest risk of recurrence were correctly identified and therefore given a higher dose, although the increase in risk was quite small. Attia et al. have found that dose > 14 Gy was significantly associated with improved PFS on multivariate analysis in atypical meningioma patients treated with radiosurgery [25].

## Conclusion

These results indicate that WHO grade III diagnosis is the strongest predictor of recurrence and mortality in aggressive meningioma patients treated with surgery and Gamma Knife. It was the only parameter significant on multivariate analysis for both recurrence and survival. Larger tumors were associated with worse survival in our series, while nuclear atypia, necrosis, and increased mitotic rate were risk factors for recurrence. This study suggests that Gamma Knife radiosurgery is a useful adjunctive therapy to surgery in the treatment of aggressive and recurrent meningiomas. The patients most likely to benefit are those with smaller tumors. This study is limited by its retrospective nature and small subject pool. Additional higher-powered studies analyzing survival and recurrence in a larger cohort are needed to identify risk factors with a smaller clinical effect.

## Abbreviations

OS: Overall survival; PFS: Progression-free survival; WHO: World Health Organization.

## Competing interests

The authors declare that they have no competing interests.

## Authors’ contributions

DF, RF, JWB, MJ and JJ contributed to collection of data, establishing of the patient database and drafting of the manuscript. TD performed critical evaluation and statistical analysis for the manuscript. MC, JD, KR, RD, IZ and JS were data collection, critical evaluation of the manuscript, and data interpretation. All authors read and approved the final manuscript.

## References

[B1] Bondy M Ligon BL Epidemiology and etiology of intracranial meningiomas: a review J Neurooncol 1996 29 197 205 10.1007/BF00165649 8858525

[B2] Longstreth WT Jr Dennis LK McGuire VM Drangsholt MT Koepsell TD Epidemiology of intracranial meningioma Cancer 1993 72 639 648 10.1002/1097-0142(19930801)72:3<639::AID-CNCR2820720304>3.0.CO;2-P 8334619

[B3] Surawicz TS McCarthy BJ Kupelian V Jukich PJ Bruner JM Davis FG Descriptive epidemiology of primary brain and CNS tumors: results from the Central Brain Tumor Registry of the United States, 1990–1994 Neuro-oncology 1999 1 14 25 11554386 10.1093/neuonc/1.1.14 PMC1919458

[B4] Porter KR McCarthy BJ Freels S Kim Y Davis FG Prevalence estimates for primary brain tumors in the United States by age, gender, behavior, and histology Neuro-oncology 2010 12 520 527 10.1093/neuonc/nop066 20511189 PMC2940648

[B5] Al-Mefty O Topsakal C Pravdenkova S Sawyer JR Harrison MJ Radiation-induced meningiomas: clinical, pathological, cytokinetic, and cytogenetic characteristics J Neurosurg 2004 100 1002 1013 10.3171/jns.2004.100.6.1002 15200115

[B6] Riemenschneider MJ Perry A Reifenberger G Histological classification and molecular genetics of meningiomas Lancet Neurol 2006 5 1045 1054 10.1016/S1474-4422(06)70625-1 17110285

[B7] Alexiou GA Gogou P Markoula S Kyritsis AP Management of meningiomas Clin Neurol Neurosurg 2010 112 177 182 10.1016/j.clineuro.2009.12.011 20056312

[B8] Whittle IR Smith C Navoo P Collie D Meningiomas Lancet 2004 363 1535 1543 10.1016/S0140-6736(04)16153-9 15135603

[B9] Durand A Labrousse F Jouvet A Bauchet L Kalamaridès M Menei P Deruty R Moreau JJ Fèvre-Montange M Guyotat J WHO grade II and III meningiomas: a study of prognostic factors J Neurooncol 2009 95 367 375 10.1007/s11060-009-9934-0 19562258

[B10] Chamberlain MC Barnholtz-Sloan JS Medical treatment of recurrent meningiomas Expert Rev Neurother 2011 11 1425 1432 10.1586/ern.11.38 21955199

[B11] Marta GN Correa SFM Teixeira MJ Meningioma: review of the literature with emphasis on the approach to radiotherapy Expert Rev Anticancer Ther 2011 11 1749 1758 10.1586/era.11.162 22050024

[B12] Bloch O Kaur G Jian BJ Parsa AT Barani IJ Stereotactic radiosurgery for benign meningiomas J Neurooncol 2012 107 13 20 10.1007/s11060-011-0720-4 22006176

[B13] Yang S-Y Park C-K Park S-H Kim DG Chung YS Jung H-W Atypical and anaplastic meningiomas: prognostic implications of clinicopathological features J Neurol Neurosurg Psychiatr 2008 79 574 580 10.1136/jnnp.2007.121582 17766430

[B14] Perry A Scheithauer BW Stafford SL Lohse CM Wollan PC “Malignancy” in meningiomas: a clinicopathologic study of 116 patients, with grading implications Cancer 1999 85 2046 2056 10223247 10.1002/(sici)1097-0142(19990501)85:9<2046::aid-cncr23>3.0.co;2-m

[B15] Pasquier D Bijmolt S Veninga T Rezvoy N Villa S Krengli M Weber DC Baumert BG Canyilmaz E Yalman D Szutowicz E Tzuk-Shina T Mirimanoff RO Rare Cancer Network Atypical and malignant meningioma: outcome and prognostic factors in 119 irradiated patients. A multicenter, retrospective study of the Rare Cancer Network Int J Radiat Oncol Biol Phys 2008 71 1388 1393 10.1016/j.ijrobp.2007.12.020 18294779

[B16] Mawrin C Perry A Pathological classification and molecular genetics of meningiomas J Neurooncol 2010 99 379 391 10.1007/s11060-010-0342-2 20809251

[B17] Engenhart-Cabillic R Farhoud A Sure U Heinze S Henzel M Mennel H-D Bertalanffy H Clinicopathologic features of aggressive meningioma emphasizing the role of radiotherapy in treatment Strahlenther Onkol 2006 182 641 646 10.1007/s00066-006-1555-3 17072521

[B18] Perry A Stafford SL Scheithauer BW Suman VJ Lohse CM Meningioma grading: an analysis of histologic parameters Am J Surg Pathol 1997 21 1455 1465 10.1097/00000478-199712000-00008 9414189

[B19] Hardesty DA Wolf AB Brachman DG McBride HL Youssef E Nakaji P Porter RW Smith KA Spetzler RF Sanai N The impact of adjuvant stereotactic radiosurgery on atypical meningioma recurrence following aggressive microsurgical Resection J Neurosurg 2013 119 475 481 10.3171/2012.12.JNS12414 23394332

[B20] Ojemann SG Sneed PK Larson DA Gutin PH Berger MS Verhey L Smith V Petti P Wara W Park E McDermott MW Radiosurgery for malignant meningioma: results in 22 patients J Neurosurg 2000 93 Suppl 3 62 67 11143265 10.3171/jns.2000.93.supplement

[B21] Stafford SL Pollock BE Foote RL Link MJ Gorman DA Schomberg PJ Leavitt JA Meningioma radiosurgery: tumor control, outcomes, and complications among 190 consecutive patients Neurosurgery 2001 49 1029 1037 discussion 1037–1038 11846894 10.1097/00006123-200111000-00001

[B22] Harris AE Lee JYK Omalu B Flickinger JC Kondziolka D Lunsford LD The effect of radiosurgery during management of aggressive meningiomas Surg Neurol 2003 60 298 305 discussion 305 10.1016/S0090-3019(03)00320-3 14505844

[B23] Huffmann BC Reinacher PC Gilsbach JM Gamma knife surgery for atypical meningiomas J Neurosurg 2005 102 Supp 283 286 15662826 10.3171/jns.2005.102.s_supplement.0283

[B24] Kondziolka D Mathieu D Lunsford LD Martin JJ Madhok R Niranjan A Flickinger JC Radiosurgery as definitive management of intracranial meningiomas Neurosurgery 2008 62 53 58 discussion 58–60 10.1227/01.NEU.0000311061.72626.0D 18300891

[B25] Attia A Chan MD Mott RT Russell GB Seif D Daniel Bourland J Deguzman AF Ellis TL McMullen KP Munley MT Tatter SB Shaw EG Patterns of failure after treatment of atypical meningioma with gamma knife radiosurgery J Neurooncol 2012 108 179 185 10.1007/s11060-012-0828-1 22359231 PMC3794718

[B26] Mori Y Tsugawa T Hashizume C Kobayashi T Shibamoto Y Gamma knife stereotactic radiosurgery for atypical and malignant meningiomas Acta Neurochir Suppl 2013 116 85 89 23417463 10.1007/978-3-7091-1376-9_13

[B27] Tamura M Kubo K Okita R Ogura M Nakao N Uematsu Y Itakura T Hayashi M Muragaki Y Iseki H Management of non-benign meningiomas with gamma knife radiosurgery Acta Neurochir Suppl 2013 116 91 97 23417464 10.1007/978-3-7091-1376-9_14

[B28] Palma L Celli P Franco C Cervoni L Cantore G Long-term prognosis for atypical and malignant meningiomas: a study of 71 surgical cases J Neurosurg 1997 86 793 800 10.3171/jns.1997.86.5.0793 9126894

[B29] Kim Y-J Ketter R Henn W Zang KD Steudel W-I Feiden W Histopathologic indicators of recurrence in meningiomas: correlation with clinical and genetic parameters Virchows Arch 2006 449 529 538 10.1007/s00428-006-0285-3 17016718

[B30] Kim JW Kim DG Paek SH Chung H-T Myung JK Park S-H Kim YH Han JH Yang S-Y Park C-K Jung H-W Radiosurgery for atypical and anaplastic meningiomas: histopathological predictors of local tumor control Stereotact Funct Neurosurg 2012 90 316 324 10.1159/000338253 22797807

[B31] DiBiase SJ Kwok Y Yovino S Arena C Naqvi S Temple R Regine WF Amin P Guo C Chin LS Factors predicting local tumor control after gamma knife stereotactic radiosurgery for benign intracranial meningiomas Int J Radiat Oncol Biol Phys 2004 60 1515 1519 10.1016/j.ijrobp.2004.05.073 15590183

[B32] Kondziolka D Flickinger JC Perez B Judicious resection and/or radiosurgery for parasagittal meningiomas: outcomes from a multicenter review. Gamma knife Meningioma Study Group Neurosurgery 1998 43 405 413 discussion 413–414 10.1097/00006123-199809000-00001 9733295

